# Simultaneous Confocal Scanning Laser Ophthalmoscopy Combined with High-Resolution Spectral-Domain Optical Coherence Tomography: A Review

**DOI:** 10.1155/2011/743670

**Published:** 2011-11-01

**Authors:** Verônica Castro Lima, Eduardo B. Rodrigues, Renata P. Nunes, Juliana F. Sallum, Michel E. Farah, Carsten H. Meyer

**Affiliations:** ^1^Retina Service, Department of Ophthalmology, Federal University of São Paulo, 04021-001 São Paulo, SP, Brazil; ^2^Department of Ophthalmology, University of Bonn, 53012 Bonn, Germany

## Abstract

We aimed to evaluate technical aspects and the clinical relevance of a simultaneous confocal scanning laser ophthalmoscope and a high-speed, high-resolution, spectral-domain optical coherence tomography (SDOCT) device for retinal imaging. The principle of confocal scanning laser imaging provides a high resolution of retinal and choroidal vasculature with low light exposure. Enhanced contrast, details, and image sharpness are generated using confocality. The real-time SDOCT provides a new level of accuracy for assessment of the angiographic and morphological correlation. The combined system allows for simultaneous recordings of topographic and tomographic images with accurate correlation between them. Also it can provide simultaneous multimodal imaging of retinal pathologies, such as fluorescein and indocyanine green angiographies, infrared and blue reflectance (red-free) images, fundus autofluorescence images, and OCT scans (Spectralis HRA + OCT; Heidelberg Engineering, Heidelberg, Germany). The combination of various macular diagnostic tools can lead to a better understanding and improved knowledge of macular diseases.

## 1. Introduction

With the advent of novel technologies, both optical coherence tomography (OCT) and confocal scanning laser ophthalmoscopy (cSLO) have been introduced successfully into the routine clinical imaging for a wide spectrum of macular diseases. The combination of these two techniques in one instrument, which offers various subsequent advantages, including exact correlation of tomographic and topographic findings, has the potential to enhance further our understanding of disease pathogenesis, diagnosis, and patient management.

In this paper, we aimed to review the history, some of the technical aspects, and important clinical applications of a high-speed, high-resolution spectral-domain OCT (SDOCT) device which also is able to combine cSLO-based fluorescein and indocyanine green angiograms, infrared, blue reflectance (“red-free”), and fundus autofluorescence (FAF) images.

## 2. History

Based on the pioneering work of Webb et al. [1, 2], confocal scanning laser ophthalmoscopes (cSLOs) have been developed for clinical use. Although limited by the optical properties of the human eye, they are able to achieve high-contrast images of the posterior segment. Today most scanning laser systems record their images in real time with a fast frame rate.

Optical coherence tomography was initially reported by Huang and coworkers in 1991 [3] and has had a tremendous subsequent impact on *in vivo* imaging of retinal diseases. It has evolved as a noninvasive technique and allows for visualization of microstructural alterations of the retinal tomographic architecture. This imaging modality is now used widely by ophthalmologists for a range of indications and has become a standard diagnostic technique [4–16]. It provides images analogous to ultrasonography, but, instead of sound, it uses light waves to obtain a reflectivity profile of the tissue under investigation, measuring the time delay and magnitude of backscattered or reflected light by low-coherence interferometry.

The OCT technique available most widely in clinical practice is referred to as time-domain OCT, because the depth information of the retina is acquired as a sequence of samples over time. This can be performed either in longitudinal cross-sections perpendicular to or in the coronal plane parallel to the retinal surface. Recently, major advances have been made regarding the image resolution—notably the development of a high-resolution OCT—and in imaging speed, signal-to-noise ratio, and sensitivity with the introduction of an ultrahigh-resolution SDOCT [17–24]. In time-domain OCT and earlier ultrahigh-resolution OCT, reference mirrors move mechanically, limiting imaging speed. In SDOCT, the reference mirror is stationary and the OCT signal is acquired either by using a spectrometer as a detector or by varying the narrowband wavelength of the light source in time (swept source). Echo time delays of light are measured by acquiring the interference spectrum of the light signal and taking its Fourier transform [25, 26]. Increasing imaging speed allows for the acquisition of images within a fraction of second, thus minimizing motion artifacts [27]. It has also become possible to acquire three-dimensional volume OCT scans that achieve comprehensive retinal coverage [28]. 

**Figure 1:**
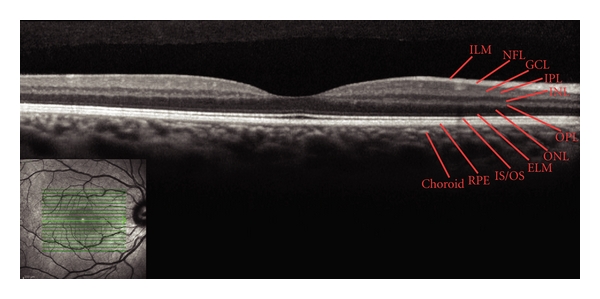
Example of a normal eye imaged with the *Spectralis* SDOCT. The infrared reflectance cSLO image (lower left) shows a normal fundus which corresponds to the normal SDOCT B-scan. The green lines represent the location, and the green arrow shows the exact orientation of the B-scan. All the retinal layers are indicated on the SDOCT scan (ILM: internal limiting membrane; NFL: nerve fiber layer; GCL: ganglion cell layer; IPL: inner plexiform layer; INL: inner nuclear layer; OPL: outer plexiform layer; ONL: outer nuclear layer; ELM: external limiting membrane; IS/OS: photoreceptor inner/outer segment junction; RPE: retinal pigment epithelium).

The combination of OCT and cSLO in one instrument offers a number of subsequent advantages, including an accurate correlation of tomographic with topographic architecture of the retina, which opens new insights in the pathogenesis and morphological alterations of retinal diseases [29, 30]. Additionally a multimodality imaging system provides a complete view of the vitreous, retina and choroid, enabling clinicians to combine information from six different modes to assess the eye: fluorescein and indocyanine green angiographies, FAF, infra-red, red-free, and SD-OCT. Simultaneous high-resolution fundus imaging and SD-OCT with the *Spectralis* (Heidelberg Engineering, Heidelberg, Germany) offer high-quality images with the certainty of knowing the location, leading to a significantly better diagnosis and monitoring of the patient (Figure 1).

## 3. Technical Aspects

The imaging system cSLO/OCT (*Spectralis* HRA+OCT) combines high speed, high-resolution SDOCT images with simultaneous recording of fluorescein and indocyanine-green angiographies, digital infrared and blue reflectance, or FAF images. On the other hand, the *Spectralis* OCT has only two modes—SDOCT and infrared. Both SDOCT devices use a new proprietary eye-tracking technology, which locks onto a specific location on the retina and relocates the site at later exams to enhance the monitoring of disease progression and treatment decisions. For image clarity, the proprietary Heidelberg Noise Reduction feature takes the axial resolution from 7 microns to 3.5 microns. And the device dual-beam imaging captures a reference scan and cross-section simultaneously for reliably accurate registration.

Regarding the technical parameters of the SDOCT, 40 000 A-scans are acquired per second using an optical resolution of approximately 7 mm in depth (axial resolution) and 14 mm transversally (lateral optical resolution). The centre wavelength of the OCT light source is typically between 870 and 880 nm. OCT scans can be recorded simultaneously with fluorescein angiography, indocyanine-green angiography, FAF, infrared, and blue reflectance images. For the A-scans the scan depth is 1.8 mm/512 pixels, providing a digital axial resolution of 3.5 *μ*m/pixel. *Spectralis* scans 100 times faster than time-domain OCT and 40% faster than most other SDOCT instruments. B-scans cover a transversal range of 15, 20, or 30° field of view. In the high-speed mode scan widths are 384, 512, and 768 A-scans per B-scan with a lateral digital resolution of 11 *μ*m/pixel and a scan rate of 89, 69 and 48 B-scans/second, respectively. In the high-speed mode, the vertical presentation of the OCT scan is magnified twice; therefore morphological alterations are presented disproportionately high in the vertical dimension. The accelerated imaging speed allows the acquisition of an image within a fraction of second, thus minimizing motion artefacts. Vertical and horizontal OCT scans are placed in the area of interest. 

The high-resolution modes encompass a scan width of 768, 1024, and 1.536 A-scans per B-scan with a lateral digital resolution of 5 *μ*m/pixel at a scan rate of 48, 37, and 25 B-scans/second, respectively. High-resolution fundus images provide clear cross-sectional scans of the retinal anatomical structure, including the retinal surface, intraretinal alterations, as well as subretinal morphologic pathologies. 

Sequences of B-scans can be acquired to image a full volume. These volume scans can be obtained at 15, 20 and 30° field of view. The number of B-scans per volume can be adjusted from 12 to 96 B-scans per 10°. In addition, it is also possible to acquire 3D volumetric OCT scans for comprehensive analysis of the entire retina and, therefore, for 3D mapping of pathologic alterations within the retinal layers including the RPE.

## 4. Clinical Applications

The new technology of the SDOCT has improved the visualization of intraretinal morphologic features allowing the evaluation of the integrity of each retinal layer and *in vivo* visualization of microstructural morphology of the retina. The high-speed, high-resolution SDOCT (*Spectralis*) has been applied over the last few years to investigate morphological substrates for alterations in eyes with various macular disorders. 

**Figure 2:**
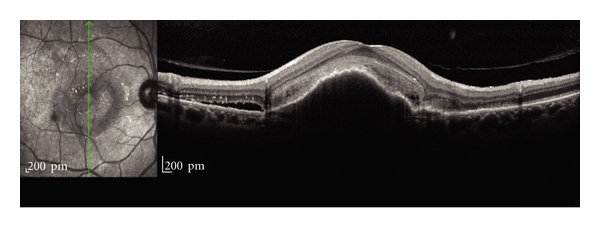
A 71-year-old man with a pigment epithelial detachment in the presence of neovascular AMD in the right eye. In the SDOCT scan the pigment epithelial detachment is readily visualized underneath the fovea associated with intraretinal cysts and subretinal fluid. It is also possible to identify the subretinal neovascular membrane and the vitreoretinal interface.

One of the most important clinical applications of this device is to help guiding diagnosis and treatment of patients with age-related macular degeneration (AMD). In early stages of AMD, drusen can be detected at their specific location [29]. Small drusen appears as localized detachments of the retinal pigment epithelium (RPE) with intact layered architecture of the photoreceptor segments. In large drusen, RPE elevation and derangement can be seen in association with a disrupted photoreceptor band. Additionally in cases of reticular drusen, another example of early AMD, the SDOCT scan shows alterations in the outer retinal and RPE layers. These ultrastructural characteristics may allow distinguishing subclasses of drusen and may allow identifying biomarkers for disease severity or risk of progression [31]. In cases of dry AMD with geographic atrophy, SDOCT scans also can confirm loss of the RPE monolayer along with atrophy of the outer neurosensory retinal layers. In most eyes with geographic atrophy the inner retinal layers are unchanged, whereas the outer retinal layers show alterations in all eyes. SDOCT provides adequate resolution for quantifying photoreceptor loss and allows visualization of reactive changes in the RPE cells in the junctional zone of geographic atrophy [30, 32, 33]. Finally SDOCT scans can be useful to delimitate and better visualize areas of pigment epithelium detachments (PEDs) and choroidal neovascular membranes in cases of wet or exudative AMD (Figure 2). The combination of the FA and the SDOCT with high-resolution and real-time mean image elaboration may enhance the detailed visualization of activity in new choroidal neovascularization, such as presence of subretinal, intraretinal, or sub-RPE fluid, intraretinal cysts, or a combination of them [34, 35]. The high sensitivity on neovascular activity expressed by the SDOCT features may be helpful in clinical practice, reducing the need of angiographies for treatment decisions [36]. 

It is well known that OCT imaging in patients with diabetic macular edema is able to reveal several structural changes in the retina, such as epiretinal membranes, intraretinal and subretinal fluid, and cystoid macular edema [37, 38]. It is a very useful tool for diagnosis, especially in challenging cases, and treatment followup. Only a limited number of studies using different SDOCT devices for assessing diabetic macular edema and diabetic retinopathy have been published [39–42]. In cases of diabetic macular edema, SDOCT has enabled us to analyze with more details the integrity of the outer retinal layers, which includes the external limiting membrane, the photoreceptors junction, the RPE, and Bruch's membrane. One first report using the *Spectralis* SDOCT showed the importance of the integrity of the external limiting membrane and the photoreceptors junction as a prognostic feature of visual improvement after treatment for diabetic macular edema [42]. 

Spectral-domain OCT has dramatically improved the visualization of the vitreomacular interface and posterior hyaloid membrane and has become a very important tool for the diagnosis and followup of patients with alterations of the vitreoretinal interface. In cases of epiretinal membranes and macular pucker, vitreoretinal adhesions at the peak elevation and retinal wrinkling can be seen in the cSLO image. In the correspondent SDOCT cross-sectional image the wrinkling of the inner retinal surface and thickening of the neurosensory retina, particularly pronounced in the outer nuclear layer and the innermost neurosensory layers, can be observed [29]. Also, in cases of macular hole, besides other features that have been well described, SDOCT can demonstrate disruption of photoreceptors junction and imaging this structure is a method of assessing structural integrity of the photoreceptors before and after macular hole surgery [43]. 

The *Spectralis* SDOCT is a very useful tool for other macular pathologies, such as retinal vascular occlusive diseases with macular involvement, central serous chorioretinopathy, macular dystrophies, idiopathic perifoveal telangiectasia, and chloroquine retinopathy. There are few studies published in the literature showing its clinical applications. Important advantages with clinical significance of this new technology compared to the time-domain technology are the ability to better visualize the vitreoretinal interface and outer retinal layers, especially the photoreceptors junction, and the possibility to obtain 3-dimensional scans allowing to image structural changes of the vitreoretinal interface and the retina in large areas. 

One advantage of the *Spectralis* OCT is the improved visualization of the choroid. Margolis and Spaide [44] described the measurement of the choroidal thickness using the enhanced depth imaging technique. It is described by positioning the device close enough to the eye to acquire an inverted image within a 5 × 30-degree area centered at the fovea and then performing manual measurements from the outer border of the RPE to the inner scleral border. In the normal studied eyes, the subfoveal choroid was thickest and grew thinner at more peripheral measurement points. Also choroidal thickness demonstrated a negative correlation with age. In different reports using the same technique, the author reported enhanced depth imaging of choroidal changes underneath a pigmented epithelial detachment in patients with exudative AMD, thus describing a novel disease entity termed age-related chorioretinal atrophy [45, 46]. The ability to visualize and quantify choroidal thickness is a very interesting area of research and may be limited to a few SDOCT devices which can overcome the technical limitations of imaging deeper structures such as analog-to-digital conversion, wavelength-dependent light scattering, and signal loss in the image path before Fourier transformation.

In conclusion, the combined cSLO/OCT system allows simultaneous recording and interpolation of topographic and tomographic images. Different cSLO imaging modes including infrared and blue reflectance, FAF and fluorescein or ICG angiography can be combined with simultaneous acquisition of OCT. This multimodality combination allows for a better understanding of the pathogenesis of several macular pathologies and improvement of diagnosis and management of patients with macular diseases.
